# Potential of miR-21 to Predict Incomplete Response to Chemoradiotherapy in Rectal Adenocarcinoma

**DOI:** 10.3389/fonc.2020.577653

**Published:** 2020-10-27

**Authors:** Susana Ourô, Cláudia Mourato, Sónia Velho, André Cardador, Marisa P. Ferreira, Diogo Albergaria, Rui E. Castro, Rui Maio, Cecília M. P. Rodrigues

**Affiliations:** ^1^Surgical Department, Hospital Beatriz Ângelo, Loures, Portugal; ^2^NOVA Medical School, Lisbon, Portugal; ^3^Research Institute for Medicines (iMed.ULisboa), Faculty of Pharmacy, Universidade de Lisboa, Lisbon, Portugal

**Keywords:** rectal cancer, chemoradiotherapy response, tumor regression grade, miR-21, biomarkers

## Abstract

**Background:** Patients with locally advanced rectal adenocarcinoma (LARC) are treated with neoadjuvant chemoradiotherapy (CRT). However, biomarkers for patient selection are lacking, and the association between miRNA expression and treatment response and oncological outcomes is unclear.

**Objectives:** To investigate miRNAs as predictors of response to neoadjuvant CRT and its association with oncological outcomes.

**Methods:** This retrospective study analyzed miRNA expression (miR-16, miR-21, miR-135b, miR-145, and miR-335) in pre- and post-chemoradiation rectal adenocarcinoma tissue and non-neoplastic mucosa in 91 patients treated with neoadjuvant CRT (50.4 Gy) and proctectomy. Two groups were defined: a pathological complete responders group (tumor regression grade—TRG 0) and a pathological incomplete responders group (TRG 1, 2, and 3).

**Results:** miR-21 and miR-135b were upregulated in tumor tissue of incomplete responders comparing with non-neoplastic tissue (*p* = 0.008 and *p* < 0.0001, respectively). Multivariate analysis showed significant association between miR-21 in pre-CRT tumor tissue and response, with a 3.67 odds ratio (OR) of incomplete response in patients with higher miR-21 levels (*p* = 0.04). Although with no significance, patients treated with 5-fluorouracil (5-FU) presented reduced odds of incomplete response compared with those treated with capecitabine (OR = 0.19; 95% confidence interval (CI) 0.03–1.12, *p* = 0.05). Moreover, significant differences were seen in overall survival (OS) in relation to clinical TNM stage (*p* = 0.0004), cT (*p* = 0.0001), presence of distant disease (*p* = 0.002), mesorectal tumor deposits (*p* = 0.003), and tumor regression grade (*p* = 0.04).

**Conclusion:** miR-21 may predict response to CRT in rectal cancer (RC).

## Introduction

Colorectal cancer (CRC) is the third most prevalent neoplasia in the world, and rectal cancer (RC) corresponded to 30% of all colorectal malignancies in 2019 (1). The current treatment for patients with locally advanced rectal adenocarcinoma (LARC) is neoadjuvant chemoradiotherapy (CRT) in order to achieve downstaging, increase R0 resections, allow sphincter-sparing surgery, and decrease local recurrence (LR) (2). After neoadjuvant treatment, patients are restaged and almost 30% develop clinical complete response (cCR) with no residual tumor identified, 46–60% achieve some degree of tumor downstaging, while 30% exhibit resistance to CRT (3). Non-responders are at increased risk of disease progression and unnecessary toxicity caused by CRT.

Recent data suggest that clinical complete responders can safely undergo a conservative approach without surgery (4). By contrast, the European Society for Medical Oncology (ESMO) guidelines recommend upfront surgery in T3a-bN1 tumors if there is no evidence of involvement of the mesorectal fascia (2). Thus, pretreatment prediction of good and bad responders could be important in deciding whether the patient should or not undergo neoadjuvant CRT. Currently, although molecular heterogeneity is a well-recognized feature of most tumors, CRC patients are still treated based solely on clinical stage. The inclusion of molecular markers in a treatment algorithm could potentially stratify patients and thus allow a better choice of candidates. No biomarkers are yet validated for selection of patients for CRT.

MicroRNAs (miRNAs) are highly conserved non-coding RNAs that act as post-transcriptional regulators binding a variety of messenger RNA targets, inhibiting its translation. Although the precise biological role of many miRNAs is yet to be entirely elucidated, up to 30% of the human genome is regulated by these molecules through influence in relevant cellular functions, including stress responses, angiogenesis, metastasis, and programmed cell death (5). Carcinogenic pathways are regulated by miRNAs and their potential role in oncogenesis raised the possibility of being used as biomarkers in cancer treatment response or prediction of prognosis (6).

Although most published data is on colon cancer, some studies have addressed RC differentiating the miRNAome between these two malignancies. Moreover, specific miRNAs have been proposed as predictors of response to CRT in RC although with some inconsistent findings (7–11). These results need to be validated and are mostly related to 5-fluorouracil (5-FU)-based therapies, not much being known about miRNAs as biomarkers of response to capecitabine.

This study aimed to investigate miRNAs as predictors of pathological response to CRT in RC. Based on literature review including our own previously published data (12), five miRNAs were chosen by virtue of having been demonstrated to be potential biomarkers for CRC. Thus, miR-16, miR-21, miR-135b, miR-145, and miR-335 expression was determined and correlated with pathological response and oncological outcomes.

## Materials and Methods

### Patients and Tissue Samples

This was a retrospective study of prospectively analyzed data and samples. Patients with RC (stages I–IV, American Joint Committee on Cancer, AJCC) diagnosed between March 2013 and September 2017 in the Surgical Department of Hospital Beatriz Ângelo (Loures, Portugal) treated with long course CRT and proctectomy were eligible.

Patients had a preoperative staging with pelvic magnetic resonance (MR), thoraco-abdomino-pelvic computed tomography (CT), and endoanal ultrasound when pelvic MR was not clinically possible. Histopathological features were confirmed by pathological analysis and patients were staged according to TNM staging system (8th edition, 2017). Patients with other histological types of rectal malignancy, not submitted to CRT or surgical resection, pregnant, or under the age of 18 were excluded.

Written and signed informed consent for collection and use of biological samples was obtained from all volunteer study participants prior to sample collection. The study protocol conformed to the ethical guidelines of the 1975 Declaration of Helsinki, as reflected in a priori approval by the institution's Human Research Committee and Ethical Committee on March 13, 2017. The study was registered in the Portuguese Data Protection Agency.

### Neoadjuvant Treatment

All patients underwent neoadjuvant CRT consisting of a 2-Gy daily fraction of pelvic irradiation, 5 times a week, in a total of 50.4 Gy. Radiation was delivered with capecitabine (825 mg/m^2^/day) or 5-FU (1,000 mg/m^2^/day on days 1–5 and days 29–33). All patients except for one received more than 80% of the planned radiotherapy with a curative intent. Surgery was performed 10–12 weeks after CRT.

### Assessment of Pathological Response

Pathology specimens were graded by tumor regression grade (TRG) according to the College of American Pathologists guidelines (CAP, TNM 7th edition). TRG was assessed by two pathologists, blinded to patients clinical data, and categorized as TRG 0 (no viable tumor cells or complete response), TRG 1 (single cells or little groups of cancer cells), TRG 2 (residual cancer outgrown by fibrosis), and TRG 3 (minimal or no tumor kill with extensive residual cancer). Tissue was retrieved from formalin-fixed paraffin embedded (FFPE) samples. Histological confirmation of the biopsy samples was done by pathologist review, and neoplastic and adjacent non-neoplastic rectal tissues were differentiated based on hematoxylin and eosin (H&E) stain. A fixed amount of tissue (80 μm) across the samples was extracted for RNA isolation. Pre-CRT RC biopsies (colonoscopy) were obtained from complete and incomplete responders as well as post-CRT tumor tissues (protectomy specimen) from incomplete responders. To allow a direct comparison of RC to matched non-neoplastic rectal mucosa, we collected adjacent (>1 cm distant) non-tumor tissue in both biopsies and protectomy specimens. Two groups of patients were defined, including a pathological complete responders group (TRG 0) and a pathological incomplete responders group (TRG 1, 2, and 3).

### RNA Isolation

For total RNA isolation, pre- and post-CRT FFPE non-neoplastic and tumor rectal tissue samples were first deparaffinized with xylene (VWR International, Radnor, PA, USA) in two washing steps at 50°C. The samples were then fully homogenized into fine particles in 100% ethanol using a motor-driven grinder and centrifuged at maximum speed for 5 min. The collected pellet was rehydrated with 95% ethanol for 10 min following a new centrifugation step at maximum speed for 5 min. Then, samples were lysed with 500 μg/mL proteinase K in 100 μL of protease digestion buffer (20 mM Tris–HCl pH 8.0, 1 mM CaCl_2_ 0.5% SDS) at 55°C. Total RNA was isolated using Ribozol™ reagent (VWR International, Radnor, PA, USA) according to the manufacturer's instructions and eluted into 20 μL RNase-free water. For a better evaluation of miRNAs quantity in total RNA, the miRNA concentration was determined using Qubit™ miRNA Assay kit (Invitrogen, Thermo Fisher Scientific, Waltham, MA, USA).

### Expression Analysis by Real-Time PCR (RT-PCR)

cDNA synthesis was performed using TaqMan® Advanced miRNA cDNA synthesis kit (Applied Biosystems, Thermo Fisher Scientific, Waltham, MA, USA) according to the manufacturer's instructions. For a uniform quantification of the quantity of miRNA to be used in cDNA, 2 μL of total RNA (corresponding to 2 ng of RNA) was extended by a 3′ poly-A tailing reaction and a 5′ adaptor ligation to the mature miRNAs. miRNAs were reverse transcribed into cDNA by reverse transcription using Universal RT primers. In order to improve detection of low-expressing miRNA targets, a pre-amplification of the cDNA was performed using the Universal miR-Amp Primers and miR-Amp Master Mix to uniformly increase the amount of cDNA for each target, maintaining the relative differential expression levels. cDNA samples were stored at −20°C. Real-time polymerase chain reaction (PCR) was performed on a Quantstudio™ 7 Flex real-time PCR instrument (Applied Biosystems, Thermo Fisher Scientific, Waltham, MA, USA) with TaqMan™ Advanced microRNA Assays (Applied Biosystems, Thermo Fisher Scientific, Waltham, MA, USA) to assess the expression profile of hsa-miR-16-5p (Assay ID 477860_mir), hsa-miR-135b-5p (Assay ID 478582_mir), hsa-miR-145-5p (Assay ID 477916_mir), hsa-miR-335-5p (Assay ID 478324_mir), and hsa-miR-21-5p (Assay ID 477975_mir). All reactions were performed in duplicate.

Due to the fact that a consensual endogenous control for miR expression in rectal tissue has still not been determined, initial preliminary analyses were performed to test several miRNAs as controls. Normalization was then performed with hsa-miR-484 (Assay ID 478308_mir), identified as the most stably expressed miRNA with the lowest expression variability between samples in these patient data set when compared with mir-1228-5p, miR-345-5p, and miR-103a-3p and the small nuclear (snRNA) U6 and RNU6B, some considered controls for CRC tissues. Expression levels were calculated by the threshold cycle (2^−ΔΔCt^ method) where ΔΔCt = (Ct target miR − Ct control) sample − (Ct target miR − Ct control) median, when amplification values were detected in the real-time PCR. Due to lack of amplification values detected by the real-time PCR in all patient tissues, a variable number of samples were included in each miRNA expression profile.

### Statistical Analysis

The estimated sample size was 86 patients (43 patients per group of low and high miR expression). Sample size was calculated with an estimated proportion of patients TRG 0 with high and low miR-21 expression of 0.067 and 0.35, respectively. Type I and II errors were set at α = 0.05 and β = 0.2, respectively. miRNA expression was analyzed using the GraphPad Prism software package, version 7.0 (GraphPad software Inc., San Diego, CA, USA). Normal distribution was determined using the D'Agostino and Pearson omnibus test. Data was analyzed according to normality of values distribution using the one-way analysis of variance (ANOVA) followed by Kruskal–Wallis non-parametric Dunn's multiple comparison test or ANOVA Tukey's multiple comparisons test according to Gaussian distribution.

Receiver operating characteristic curve (ROC) analysis was then conducted, establishing the optimal cutoffs for each miRNA before CRT in non-neoplastic and tumor tissue, determined as the point closest to the top left part of the plot with perfect sensibility and sensitivity. All miRNAs were dichotomized according to these cutoffs. Further analysis was also performed to explore the best discriminative cutoff point for miR-21 by comparing the cutoff determined in this study (1.18) with the previously reported miR-21 cutoff (2.8) (13). Both cutoffs presented a similar area under the curve (AUC), with our cutoff having an AUC value of 0.65 (95% CI = 0.518–0.790), a higher specificity (66 vs. 60%), a lower sensitivity (64 vs. 87%), a similar positive predictive value (PPV) (92 vs. 90%) and a lower negative predictive value (NPV) (29 vs. 43%) (Supplementary Figure 1 and Supplementary Table 1). Although both dichotomizations presented similar performance, we chose the cutoff determined in this study that yielded a better-distributed categorization of miR-21.

Simple and multiple logistic regressions were used to correlate each variable with the outcome response after CRT: “pathological complete response (TRG 0)” or “pathological incomplete response (TRG 1, 2, and 3).” For continuous variables, linearity of the logit in the predictor was assessed using a cubic spline and Wald test of linearity.

The association between high and low miR-21 expression and clinical characteristics was tested with chi-square test. Only variables with *p* ≤ 0.25 in simple logistic regression or considered clinically relevant were selected to multiple logistic regression. Multicollinearity was also analyzed through the observation of variance inflation factors. A stepwise both-selection technique was used to create the multiple regression model. ROC curve was computed and the respective AUC was calculated to assess discriminatory ability of the model.

## Results

### Patient Clinical Parameters

Demographic and clinical parameters of the 91 patients are summarized in Table 1. With 4 patients lost (4.4%), median follow up was 4.2 years.

**Table 1:** Patient clinical parameters.

**Clinical parameters**		**Patients (*n* = 91)**
Gender, *n* (%)	Male	60 (66)
	Female	31 (34)
Age, median		68 (45–83)
BMI, median		26 (15–45)
ASA score, *n* (%)	Not discriminated	11 (12)
	I	2 (2)
	II	56 (62)
	III	21 (23)
	IV	1 (1)
Grade	G1/G2	85 (93)
	G3/G4	6 (7)
Location (%)	1/3 superior	19 (21)
	1/3 medium	28 (31)
	1/3 inferior	44 (48)
Tumor extension (mm), median		58 (5–120)
Distance to anal verge (mm), median		60 (0–130)
cT	1	1 (1)
	2	10 (11)
	3	64 (70)
	4	16 (18)
cN	0	9 (10)
	+	82 (90)
cM	0	78 (86)
	1	13 (14)
CRM, *n* (%)	Free	67 (74)
	Threatened or invaded	24 (26)
EMVI, *n* (%)	Negative	86 (95)
	Present	5 (5)
c Stage, *n* (%)	I	3 (3)
	II	8 (9)
	III	68 (75)
	IV	12 (13)
CEA (mg/mL)		1.9 (0.5–163)
Chemotherapy	Capecitabine based	83 (91)
	5-FU based	8 (9)
TRG (CAP), *n* (%)	0	15 (17)
	1	24 (26)
	2	33 (36)
	3	19 (21)

*BMI, Body Mass Index; ASA, American Society of Anaesthesiologists; CRM, circumferential resection margin; EMVI, extramural vascular invasion; CEA, carcinoembrinonary antigen; TRG, tumor regression grade; CAP, College of American Pathologists*.

### miRNA Expression in Complete and Incomplete Responders

**Figure 1:**
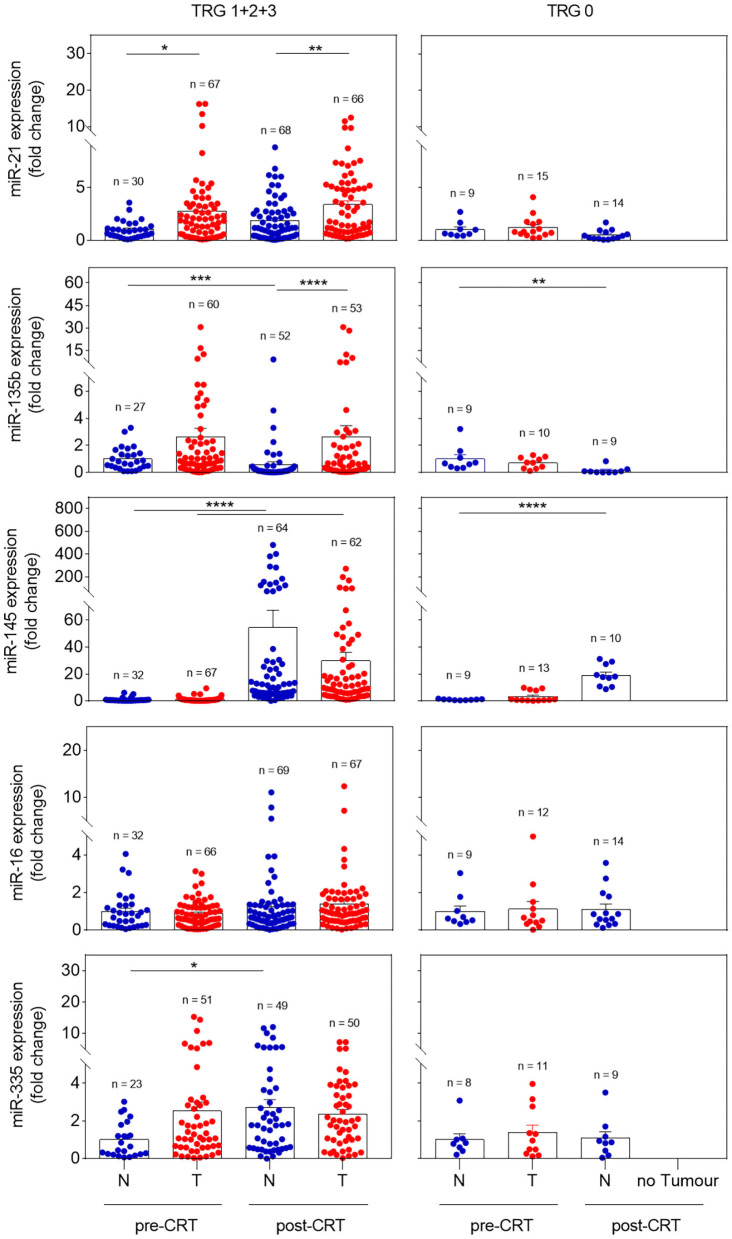
Expression profile of miR-21, miR-135b, miR-145, miR-16, and miR-335 in pre- and post-CRT non-neoplastic and tumor tissues in incomplete (TRG 1 + 2 + 3) and complete responders (TRG 0). Pre-CRT non-neoplastic tissue samples used in this study were derived from a maximum of 37 and 10 patients in TRG 1 + 2 + 3 and TRG 0 groups, respectively. Pre-CRT tumor tissue and post-CRT tissue samples were analyzed from a maximum of 76 patients (TRG 1 + 2 + 3) and 15 patients (TRG 0). Data are mean ± SEM (*p < 0.05, **p < 0.01, ***p < 0.001, ****p < 0.0001), in which N corresponds to non-neoplastic tissue and T to tumor tissue.

miRNA expression profiles were analyzed in non-neoplastic and tumor rectal tissue before and after CRT in all 91 patients. Significant changes were observed when comparing incomplete and complete responders (Figure 1). In incomplete responders, miR-21 revealed higher expression in pre-CRT tumor tissue in comparison with non-neoplastic tissue (*p* = 0.03). Post-CRT samples also presented higher levels of miR-21 in tumor tissue (*p* = 0.008). In contrast, in complete responders, miR-21 showed similar levels in pre-CRT tumor and non-neoplastic tissue.

miR-135b presented a profile equivalent to miR-21. In incomplete responders, miR-135b upregulation was detected in tumor tissue, either pre- or post-CRT (*p* < 0.0001), whereas in complete responders equal levels were found in pre-CRT tumor samples and non-neoplastic tissue. Although miR-145 expression showed significant differences among pre- and post-CRT non-neoplastic and tumor tissues (*p* < 0.0001) in incomplete responders, similar results were detected in complete responders, suggesting a lack of discriminative value of this miRNA.

Moreover, there were no significant differences in miR-16 and miR-335 expression between groups. Thus, these results suggest that miR-21 and miR-135b might be useful biomarkers to predict treatment response.

### Identification of miRNAs Involved in TRG

The significantly different expression of miRNAs between incomplete (TRG 1, 2, and 3) and complete responders (TRG 0) suggested a possible association between miRNA expression and treatment response. The relation between miRNA in pre-CRT samples and response was analyzed with logistic regression (Table 2). A significant association was found between miR-21 in pre-CRT tumor tissue and TRG. Patients with expression higher than 1.18 (fold change) were 3.58 more likely to obtain an incomplete response than those with expression lower than 1.18 (*p* = 0.03). However, there was no association between pre-CRT non-neoplastic or tumor tissue expression of miR-135b and TRG. The same was found for miR-16, miR-145, and miR-335. Given the association of miR-21 and response, we proceeded with the study of this miRNA.

**Table 2:** Association between miRNA expression and TRG.

**Variables**		**OR**	**95% CI**	***p*-Value**
miR-21	≤0.66	1.00		
Pre-CRT non-neoplastic	>0.66	1.428	0.32–6.79	0.6407
miR-21	≤1.18	1.00		
Pre-CRT tumor	>1.18	3.58	1.13–12.65	0.0346
miR-135b	≤0.8	1.00		
Pre-CRT non-neoplastic	>0.8	1.85	0.40–10.27	0.4420
miR-135b	≤1.01	1.00		
Pre-CRT tumor	>1.01	2.33	0.58–11.62	0.25
miR-145	≤1.28	1.00		
Pre-CRT non-neoplastic	>1.28	0.65	0.11–5.18	0.643
miR-145	≤0.73	1.00		
Pre-CRT tumor	>0.73	0.88	0.26–3.02	0.838
miR-16	≤0.77	1.00		
Pre-CRT non-neoplastic	>0.77	2.00	0.44–10.80	0.3806
miR-16	≤0.54	1.00		
Pre-CRT tumor	>0.54	1.75	0.49–6.19	0.375
miR-335	≤1.16	1.00		
Pre-CRT non-neoplastic	>1.16	4.5	0.64–91.58	0.191
miR-335	≤1.01	1.00		
Pre-CRT tumor	>1.01	1.86	0.49–7.24	0.354

*Simple logistic regression using miRNA dichotomized according to cut-offs determined with ROC curve analysis. OR, odds ratio of incomplete/non-response; CI, confidence interval*.

### Clinical Parameters and TRG in miR-21 Expressing Patients

From the initial group of 91 patients, only 82 patients expressed miR-21 due to lack of amplification. Although with no significant association between type of radio-sensitizing agent and TRG, patients treated with 5-FU presented reduced odds ratio (OR) of incomplete response compared with patients treated with capecitabine [OR = 0.19; 95% confidence interval (CI) 0.03–1.12, *p* = 0.05]. It was also recognized a definitive trend toward reduced odds of incomplete response with longer waiting times (OR = 0.87; 95% CI 0.73–1.01, *p* = 0.08). However, there was no association between patient gender, age, weigh, American Society of Anaesthesiologists (ASA) score, body mass index (BMI), tumor location, tumor extension, histological grade, pre-therapeutic carcinoembrionary antigen (CEA), radiological involvement of the circumferential resection margin (CRM), presence of extramural vascular invasion (EMVI), mesorectal deposits (N1c), extramesorectal nodes, cT, cN, cM, stage (TNM, AJCC), and TRG (Table 3).

**Table 3:** Clinical parameters and TRG in miR-21 expressing patients.

**Simple logistic regression**		**TRG 0 *n* = 15**	**TRG 1 + 2 + 3 *n* = 67**	**OR**	**95% CI**	***p*-Value**
**Continuous variables**		**Median (Max–Min)**	**Median (Max–Min)**			
Age		67.0 (53–81)	68 (45.0–83)	1.00	0.94–1.06	0.976
Weight		70.0 (45–113)	68 (44.0–119)	0.99	0.96–1.03	0.645
BMI		25.0 (19–41)	26 (15.0–45)	1.00	0.91–1.13	0.921
Tumor extension (mm)		54.5 (21–110)	56 (5–120)	0.99	0.97–1.03	0.901
CEA		2.8 (0.5–8.3)	1.9 (0.5–163)	1.07	0.99–1.29	0.299
Weeks post-chemo		11 (7.0–28)	10 (2.0–21)	0.87	0.73–1.01	0.081
**Categorical variables**		**Number**	**Number**			
Gender	Male	11	45	1.00		
	Female	4	22	1.34	0.41–5.29	0.643
Tumor location	0	3	14	1.00		
	1	8	16	0.43	0.08–1.81	0.271
	2	4	37	1.98	0.35–10.13	0.407
ASA	1 + 2	9	54			
	3 + 4	6	13	0.36	0.11–1.24	0.0955
CRM MR	Free	11	50		1.00	
	Threatened	1	4	0.88	0.12–18.11	0.913
	Invaded	3	13	0.95	0.25–4.66	0.947
Extramesorectal nodes	Negative	12	43	1.00		
	Positive	3	24	2.23	0.63–10.50	0.247
cT	1 + 2	1	8	1.00		
	3 + 4	14	59	0.53	0.03–3.23	0.561
cN	0	2	6	1.00		
	1	13	61	1.56	0.21–7.721	0.608
cM	0	14	57	1.00		
	1	1	10	2.46	0.42–46.96	0.41
Stage	I	1	2	1.00		
	II	2	5	1.25	0.04–23.53	0.880
	III	11	51	2.32	0.10–26.38	0.508
	IV	1	9	4.50	0.14–156.82	0.352
Stage	I + II	3	7	1.00		
	III + IV	12	60	2.14	0.42–8.99	0.315
Chemotherapy	Capecitabine	12	64	1.00		
	5-FU	3	3	0.188	0.03–1.12	0.05

*Simple logistic regression analysis using TRG as dependent variable and clinical/molecular variables as independent variables. From the initial group of 91 patients, 82 expressed miR-21*.

*TRG, Tumor regression grade; OR, odds ratio of incomplete response; CI, confidence interval; BMI, body mass index; CEA, carcinoembrionary antigen; ASA, American Society of Anaesthesiologists; CRM, circumferential resection margin; MR, magnetic resonance*.

### Clinical Parameters and Levels of miR-21 Expression

Although no statistically significant association between clinical parameters and expression of miR-21 was observed, a near significant association was established between this miRNA and TRG, with higher proportion of incomplete response in patients with higher miR-21 levels (*p* = 0.06) (Table 4). In multivariate analysis, after adjustment for clinically and statistically relevant variables (disease stage and ASA score), this association was again demonstrated with odds of incomplete response 3.67 times greater in individuals with a miR-21 overexpression (>1.18-fold change) when compared with those with lower miR-21 levels (≤1.18-fold change) (95% CI 1.13–13.5; *p* = 0.04) (Table 5).

**Table 4:** Clinical parameters and levels of miR-21 expression.

**Variables**		**Number (%)**	**High miR-21**	**Low miR-21**	***p*-Value**
miR-21 pre-CRT tumor		82 (100)	48 (58.5)	34 (41.5)	
Age	<60	15 (18.3)	7 (14.6)	8 (23.5)	0.302
	≥60	67 (81.7)	41 (85.4)	26 (76.5)	
Sex	Male	56 (68.3)	32 (66.7)	24 (70.6)	0.707
	Female	26 (31.7)	16 (33.3)	10 (29.4)	
BMI	Low weight	1 (1.2)	0 (0)	1 (2.9)	0.236
	Normal	27 (32.9)	17 (35.4)	10 (29.4)	
	Pre-obesity	39 (47.6)	25 (52.1)	14 (41.2)	
	Obesity	15 (18.3)	6 (12.5)	9 (26.5)	
ASA score	1	2 (2.4)	1 (2.1)	1 (2.9)	0.330
	2	53 (64.6)	29 (60.4)	24 (70.6)	
	3	18 (22)	11 (22.9)	7 (20.6)	
	4	1 (1.2)	1 (2.9)	0 (0)	
	ND	8 (9.8)	7 (14.6)	1 (2.9)	
Stage pre-CRT	I	3 (3.7)	1 (2.1)	2 (5.9)	0.720
	II	7 (8.5)	4 (8.3)	3 (8.8)	
	III	62 (75.6)	36 (75.0)	26 (76.5)	
	IV	10 (12.2)	7 (14.6)	3 (8.8)	
Stage post-CRT	0	12 (14.6)	6 (12.5)	6 (17.6)	0.607
	I	6 (7.3)	4 (8.3)	2 (5.9)	
	II	6 (7.3)	5 (10.4)	1 (2.9)	
	III	9 (11.0)	4 (8.3)	5 (14.7)	
	IV	3 (3.7)	1 (2.1)	2 (5.9)	
	NA	5 (6.1)	4 (8.3)	1 (2.9)	
	ND	41 (50)	24 (50.0)	17 (50.0)	
Grade pre-CRT	Low	77 (93.9)	45 (93.8)	32 (94.1)	1.00
	High	5 (6.1)	3 (6.2)	2 (5.9)	
cT	1	1 (1.2)	1 (2.1)	0 (0.0)	0.852
	2	8 (9.8)	5 (10.4)	3 (8.8)	
	3	59 (72.0)	34 (70.8)	25 (73.5)	
	4	14 (17.1)	8 (16.7)	6 (17.6)	
cN	0	8 (9.8)	4 (8.3)	4 (11.8)	0.606
	1	74 (90.2)	44 (91.7)	30 (88.2)	
cM	0	71 (86.6)	41 (85.4)	30 (88.2)	0.712
	1	11 (13.4)	7 (14.6)	4 (11.8)	
pTRG	TRG 0	15 (18.3)	5 (10.4)	10 (29.4)	0.064
	TRG 1	21 (25.6)	16 (33.3)	5 (14.7)	
	TRG 2	32 (39.0)	20 (41.7)	12 (35.3)	
	TRG 3	14 (17.1)	7 (14.6)	7 (20.6)	
Distant recurrence	No	60 (73.2)	33 (68.8)	27 (79.4)	0.283
	Yes	22 (26.8)	15 (31.2)	7 (20.6)	
Local recurrence	No	75 (91.5)	43 (89.6)	32 (94.1)	0.694
	Yes	7 (8.5)	5 (10.4)	2 (5.9)	
Death	No	61 (74.4)	33 (68.8)	28 (82.4)	0.164
	Yes	21 (25.6)	15 (31.2)	6 (17.6)	

*From the initial group of 91 patients, 82 expressed miR-21*.

*ASA, American Society of Anaesthesiologists; BMI, body mass index; CRT, chemoradiotherapy; pTRG, pathological tumor regression grade*.

**Table 5:** Association between clinical parameters and TRG.

**Variables**		**OR**	**95% CI**	***p*-Value**
Stage	1 + 2	1.00		
	3 + 4	2.16	0.388–10.16	0.341
miR-21	≤ 1.18	1.00		
	>1.18	3.67	1.126–13.49	0.036
ASA score	1 + 2	1.00		
	3 + 4	0.33	0.090–1.185	0.082

*Multiple logistic regression analysis using TRG as dependent variable and disease stage, miR-21 and ASA score as independent variables*.

*OR, odds ratio; CI, confidence interval; ASA, American Society of Anaesthesiologists*.

### Oncological Outcomes

**Figure 2:**
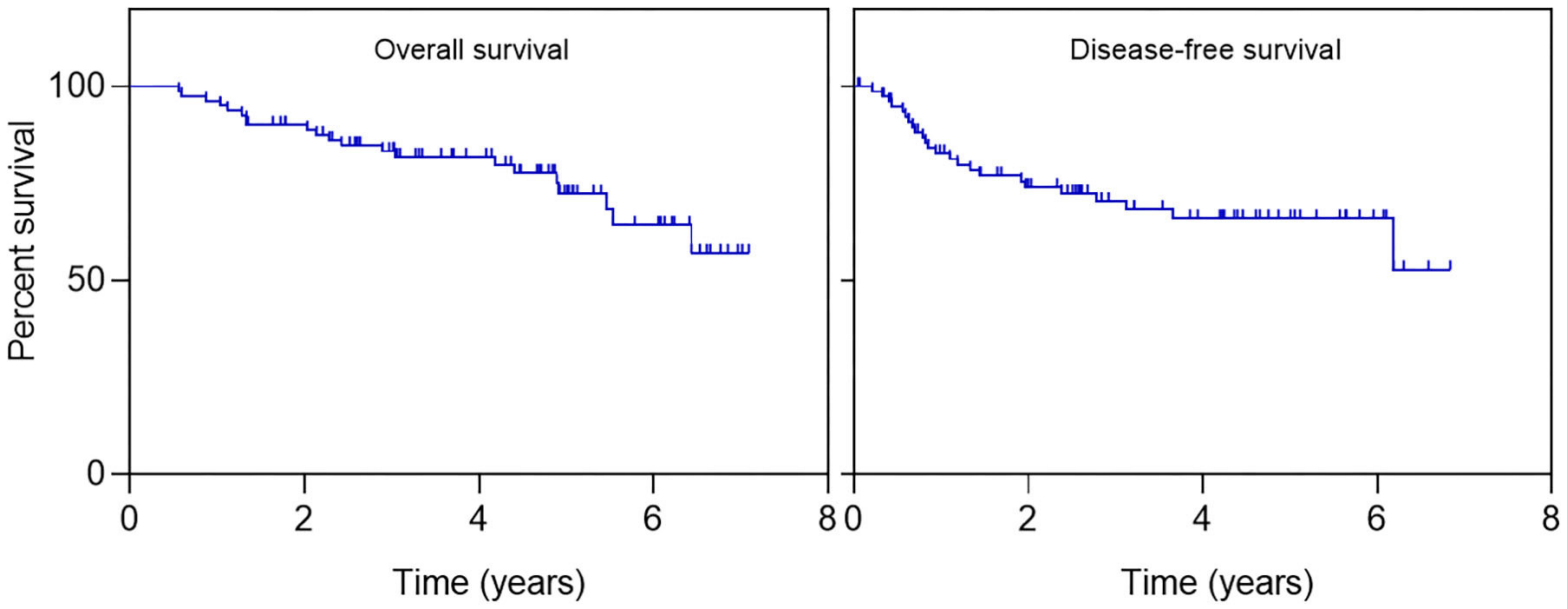
Patient outcomes in miR-21-expressing patients. Kaplan–Meier curves for overall survival and disease-free survival.

Overall survival (OS) at 2 and 5 years was 90% (95% CI 83.4–96.9) and 72% (95% CI 61.6–85.1), respectively. Overall disease-free survival (DFS) at 2 and 5 years was 74.1% (95% CI 64.4–84.8) and 66% (95% CI 55–80), respectively (Figure 2).

**Figure 3:**
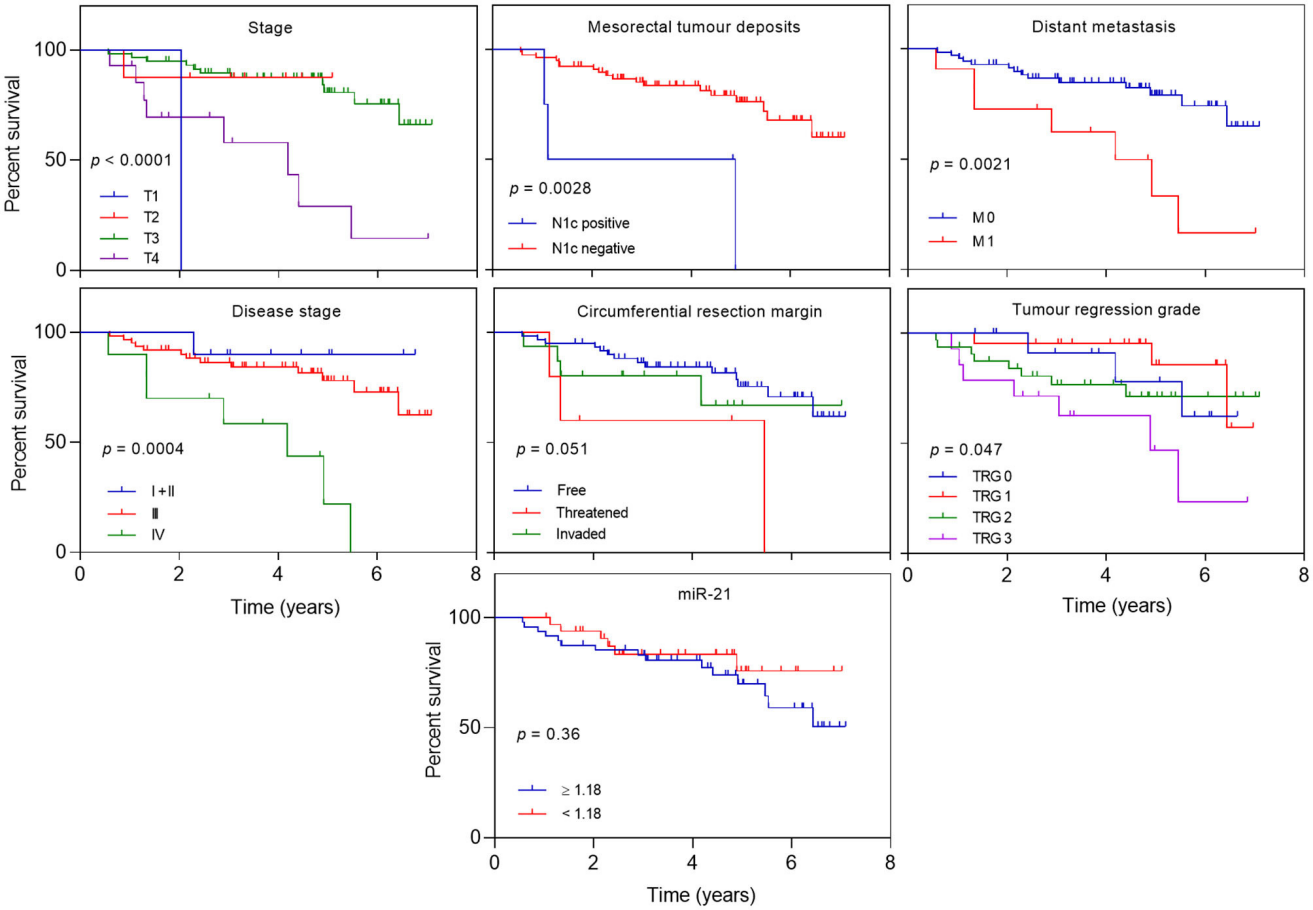
Overall survival according to clinical and oncological parameters. Kaplan–Meier curves estimating overall survival according to stage, mesorectal tumor deposits (cN1c), M, stage, circumferential resection margin (CRM) involvement, tumor regression grade and levels of miR-21.

Overall survival was not influenced by age, gender, tumor location, grade, mesorectal nodes, extramesorectal nodes, type of radio-sensitizing agent, post-operative complications, and levels of miR-21 (*p* = 0.36) (Figure 3 and Supplementary Figure 2). As expected, there was an impact in OS in relation to T (*p* < 0.0001) mesorectal tumor deposits, N1c (*p* = 0.003), distant metastasis M (*p* = 0.002), stage (*p* = 0.0004), and TRG (*p* = 0.04) with a borderline significance for threatened circumferential resection margin, CRM (*p* = 0.05) (Figure 3). Also, there was increase death risk in individuals with higher cT (HR = 4.78; 95% CI 1.96–11.66, *p* = 0.0006), higher stage (HR = 11.1; 95% CI 1.34–91.88, *p* = 0.03), threatened mesorectal fascia (HR = 4.24; 95% CI 1.19–15.08, *p* = 0.03), positive N1c (HR = 5.47; 95% CI 1.56–19.14, *p* = 0.008), distant metastasis (HR = 3.78; 95% CI 1.52–9.4, *p* = 0.004), and TRG 3 (HR = 3.25; 95% CI 0.83–12.71, *p* = 0.08). No association was, however, established between miR-21 expression and risk of death (Table 6).

**Table 6:** Patient survival according to miR-21 expression and clinical parameters.

		**Patients *n* = 82**	**Deaths *n* = 21**	**Survival**		**Simple cox proportional hazards models**			
				**Mean**	***p-*Value**	**Coef**	**HR**	**95% CI**	***p-*Value**
miR-21	<1.18	34	6	6.04			1.00		0.36
	≥1.18	48	15	5.50	0.36	0.44	1.56	0.60–4.03	
Age	<60	17	3	5.81	0.58		1.00		0.57
	>60	65	18	5.51		0.35	1.42	0.41–4.8	
Sex	Male	56	16	5.56	0.57		1.00		0.57
	Female	26	5	5.82		−0.29	0.75	0.27–2.04	
Tumor location	1/3 upper	17	3	6.09	0.14		1.00		
	1/3 middle	24	5	6.13		0.05	1.045	0.25–4.40	0.94
	1/3 lower	41	13	5.16		0.91	2.49	0.70–8.85	0.158
ASA score	1 + 2	55	14	5.71	0.97		1.00		
	3 + 4	19	5	5.44		0.10	1.11	0.39–3.094	0.879
	ND	8	2	5.10		0.12	1.12	0.25–4.99	0.986
Stage	I+II	10	1	6.32	0.0004		1.00		
	III	61	13	5.74		0.83	2.31	0.30–17.65	0.4218
	IV	11	7	3.54		2.41	11.10	1.34–91.88	0.0256
Grade	Low	77	19	5.74	0.41		1.00		
	High	5	2	4.87		0.60	1.83	0.42–7.88	0.42
CRM	Free	61	14	5.91	0.051		1.00		
	Threatened	5	3	3.77		1.45	4.24	1.19–15.08	0.025
	Invaded	16	4	5.47		0.51	1.67	0.54–5.142	0.37
EMVI	Negative	77	20	4.45	0.77		1.00		0.768
	Positive	5	1	4.20		0.31	1.36	0.17–10.41	
N1c	Negative	78	18	5.15	0.0028		1.00		0.00788
	Positive	4	3	2.98		1.69	5.47	1.56–19.14	
Extramesorectal nodes	Negative	55	13	5.77	0.26		1.00		
	Positive	27	8	5.15		0.51	1.67	0.68–4.07	0.263
cT	T1-3	68	13	6.05	0.0001		1.00		
	T4	14	8	3.73		1.56	4.78	1.96–11.66	0.0006
cN	0	8	1	6.25	0.42		1.00		
	1	74	20	4.48		0.81	2.24	0.29–16.7	0.432
cM	0	71	14	5.98	0.0021		1.00		
	1	11	7	4.02		1.33	3.78	1.52–9.4	0.00416
TRG	0	15	3	5.94	0.047		1.00		
	1	21	3	6.32		0.49	0.61	0.12–3.05	0.5504
	2	32	8	5.54		0.34	1.41	0.37–5.35	0.6130
	3	14	7	4.31		1.18	3.25	0.83–12.71	0.0897
Chemotherapy	Capecitabine	76	19	5.24	0.47		1.00		
	5-FU	6	2	4.83		0.54	1.71	0.39–7.43	0.476
Post-op complications	Negative	38	9	5.85	0.6		1.00		
	Positive	44	12	5.55		0.23	1.26	0.53–0.98	0.604

*Kaplan–Meier estimates, simple cox proportional hazards model. From the initial group of 91 patients, 82 expressed miR-21*.

*HR, hazard ratios; CI, confidence interval; ASA, American Society of Anaesthesiologists; CEA, carcinoembrionary antigen; CRM, circumferential resection margin; EMVI, extramural vascular invasion; TRG, tumor regression grade*.

Finally, the utility of miR-21 as a predictor of survival was investigated. The model of prediction, in multivariate analysis, adjusted to the most relevant clinical variables, did not show a significant association between risk of death and higher miR-21 expression (HR = 2.68; 95% CI 0.86–8.36, *p* = 0.09) (Table 7).

**Table 7:** Association between patients survival and miR-21 expression.

		**Multiple cox proportional hazards models**				**Multiple cox proportional hazards models**			
		**Coef**	**HR**	**95% CI**	***p-*Value**	**Coef**	**HR**	**95% CI**	***p-*Value**
miR-21	<1.18	Not included					1.00		
	≥1.18					0.99	2.68	0.86–8.36	0.089
Mesorectal deposits	Negative		1.00				1.00		
	Positive	1.84	6.26	1.74–22.48	0.005	2.49	12.17	2.61–56.70	0.001
cT	T1-3		1.00				1.00		
	T4	1.63	5.09	2.06–12.61	0.0004	1.69	5.45	2.17–13.63	0.0003
C-statistics		0.671				0.674			

*Multiple Cox Proportional Hazards Models obtained with stepwise variable selection*.

*HR, hazard ratios; CI, confidence interval*.

## Discussion

Rectal cancer (RC) patients treated with CRT urgently need biomarkers to distinguish responders from non-responders and allow individualized treatment, with non-responders avoiding neoadjuvant therapy and complete responders eluding mutilating resections. In this work, we investigated five miRNAs as biomarkers to predict response to CRT in RC.

miR-145 and miR-335 are acknowledged to act as tumor suppressor genes (14, 15) and miR-145 is overexpressed in post-CRT tumor tissue in comparison with pre-CRT with significant correlation with tumor regression (7). In our work, no differences were detected in these miRNAs before and after CRT and no correlation was found with response. In addition, miR-16 has been described as a tumor suppressor with downregulation predicting poor prognosis in CRC (16). In our study, miR-16 was not a predictor of response either. miR-135b is an oncomiR that often mediates CRC genes whose overexpression has been correlated with tumor stage and poor clinical outcome (17). We have further analyzed its potential as predictor of response to CRT and found significant differences in expression. In incomplete responders, higher miR-135b levels were found in both pre- and post-CRT tumor tissues comparing with non-neoplastic tissues, whereas in complete responders similar expression was obtained in all samples. We could not, however, correlate miR-135b expression with clinical parameters or TRG.

Finally, in our study we found that incomplete responders had higher miR-21 expression in tumor tissue in comparison with non-neoplastic tissue in both pre- and post-CRT samples. In contrast, complete responders had similar levels in all samples. Moreover, an association was discovered between pre-CRT tumor miR-21 levels and TRG, with a 3.67 odds of non-response in patients with expression higher than 1.18 (*p* = 0.04). Higher miR-21 expression in the tumor prior to treatment was indicative of a worst response. As expected, OS was influenced by cT, cM, N1c, TRG, and threatened CRM but no association was noted between risk of death and miR-21 expression. Thus, in this study, we showed that miR-21 expression levels before neoadjuvant therapy had the potential to predict response and that patients with miR-21 overexpression exhibited less response to standard CRT dose. This did not, however, translate in a change in survival.

miR-21 is often upregulated in solid tumors influencing cell proliferation, invasion, and apoptosis (18). Considered to be an oncomiR, multiple studies report its role in CRC biology as a screening, diagnostic, and prognostic biomarker (6, 19–23). Also, miR-21 upregulation has been related to advanced stage, presence of positive lymph nodes, venous invasion, and metastatic behavior (24, 25).

In contrast to colonic cancer, very limited data is available on miRNA expression and response to CRT in RC (26–28) with most patients treated with 5-FU-based therapies and not capecitabine. So far, miR-21 has been described to induce resistance to 5-FU when overexpressed in colon cancer cells (13, 29), which could eventually explain its effect regarding 5-FU-based CRT response.

Literature is controversial regarding the use of miR-21 as biomarker of response in RC. In one study with 76 RC biopsies, high pre-CRT miR-21 could discriminate responders from non-responders with an OR of 9.75 (95% CI 2.24–42) (30). Recently, 96 complete responders had significantly inferior miR-21 expression comparing with patients with incomplete response (*p* = 0.01), with an AUC of 0.669 (95% CI 0.55–0.79, *p* = 0.01) (31). These observations are in accordance with our own results and with the well-reported miR-21 oncomiR function. Contrarily, in another study, 40 RC patients treated with 5-FU-based CRT had higher miR-21 in post-CRT tumor tissue than in pre-CRT tumor and post-CRT normal tissues (7). It has also been reported overexpression of miR-21 in patients with complete response (32, 33). It is important to note, however, that in one of these studies, the responder group involved a different set of patients, including individuals submitted to surgery with pathological complete response (pCR) and patients with complete clinical response (cCR) not treated with surgery but only observed by follow up (33). The latest might have had undetectable residual disease and not be a real pCR. This different response assessment invalidates an accurate comparison of results and may explain the distinct observations when compared with our work.

Overall, the heterogeneity of results is related to the fact that most published studies included patients with colon and RC, 2 distinct entities with different treatment strategies that previous contributions failed to separate. Patient variability, nature of biological samples (blood, tissue, serum, or feces), miRNA extraction, array platforms, bioinformatics analysis, and different TRG grading systems also contribute to these discrepancies. Likewise, it is possible that population may have different miRNA signatures and transcriptome vary according to tumor site.

In this study, we recognized the significance of miR-21 expression in RC in response to neoadjuvant CRT. Although including a sizeable cohort with uniform sampling and treatment, there is a potential for intratumoral heterogeneity and results are currently being validated in a prospective series. If confirmed as a biomarker, translation to clinical practice with miR-21 inclusion in treatment algorithms may allow a stratification of responders and better selection of candidates for CRT.

Of note, in addition to possible markers of response and prognosis at the time of diagnosis, miRNAs may be potential therapeutic targets *via* reintroducing miRNAs absent in carcinogenic pathways or by inhibiting oncomiRs (34–36). Likewise, affecting miRNAs implicated in the mechanism of resistance to CRT may improve the therapeutic outcome. The biggest challenge will continue to be the identification of miRNA targets that shed light on our understanding of downstream cellular mechanisms of resistance to CRT.

In conclusion, the present study suggests miR-21 as a potential biomarker of pathological response in RC. The results provide an association between a miRNA in the neoadjuvant therapy setting and tumor regression with significant implications that strengthen the role of miRNAs as predictors of response. This work further emphasizes the need for prospectively conducted trials of miRNA as biomarkers in RC patients treated with CRT.

## Data Availability Statement

The raw data supporting the conclusions of this article will be made available by the authors, without undue reservation.

## Ethics Statement

The studies involving human participants were reviewed and approved by the institution's Ethical Committee (Comissão de Ética para a Saúde do Hospital Beatriz Ângelo) on 13th March 2017. The study was registered in the Portuguese Data Protection Agency (Comissão Nacional de Protecção de Dados) on 27th January 2017. Written and signed informed consent for collection and use of biological samples was obtained from all volunteer study participants prior to sample collection.

## Author Contributions

SO: study conception and design, funding, sample collection, sample treatment, analysis and interpretation of the data, drafting of the article, critical revision of the article for important intellectual content, and final approval of the article. CM: miRNA isolation, expression analysis, interpretation of the data, drafting of the article, critical revision of the article for important intellectual content, and final approval of the article. SV: statistical analysis of the data and final approval of the article. AC: miRNA isolation, expression analysis, interpretation of the data, and final approval of the article. MF and DA: sample collection and critical revision of the article for important intellectual content. RC and RM: critical revision of the article for important intellectual content and final approval of the article. CR: study design, funding, critical revision of the article for important intellectual content, and final approval of the article. All authors: contributed to the article and approved the submitted version.

## Conflict of Interest

The authors declare that the research was conducted in the absence of any commercial or financial relationships that could be construed as a potential conflict of interest.
