# Supplementary material for: Potential of miR-21 to Predict Incomplete Response to Chemoradiotherapy in Rectal Adenocarcinoma
Source: Front Oncol. 2020 Oct 27;10:577653. doi: 10.3389/fonc.2020.577653 (PMC7653406; doi:10.3389/fonc.2020.577653)
Supplement: Supplementary file 1 [file Data_Sheet_1.docx]

**SUPPLEMENTARY MATERIALS**

**Potential of miR-21 to predict incomplete response to chemoradiotherapy in rectal adenocarcinoma**

Susana Ourôꝉ ^1,2,^*; Cláudia Mouratoꝉ^3^, Sónia Velho^1^, André Cardador^3^, Marisa P. Ferreira^1^, Diogo Albergaria^1^, Rui E. Castro^3^, Rui Maio^1,2^ and Cecília M. P. Rodrigues^3*^

ꝉ Co-first authors

^1^Surgical Department, Hospital Beatriz Ângelo, Loures, Portugal.

^2^NOVA Medical School, Lisbon, Portugal.

^3^Research Institute for Medicines (iMed.ULisboa), Faculty of Pharmacy, Universidade de Lisbon, Portugal.

***Corresponding authors:** Susana Ourô, Hospital Beatriz Ângelo, Surgical Department, Avenida Carlos Teixeira 514, 2674-514 Loures, Portugal, [smrouro@gmail.com](mailto:smrouro@gmail.com); Cecília M. P. Rodrigues, Faculty of Pharmacy, Universidade de Lisbon, Avenida Prof. Gama Pinto, 1649-003 Lisbon, Portugal

**Figures**

**Supplementary Figure S1.** ROC curve analysis**.** Comparison of miR-21 cut-offs determined by Caramès *et al* (2.8) (30) and by this study (1.18). AUC: area under the curve.


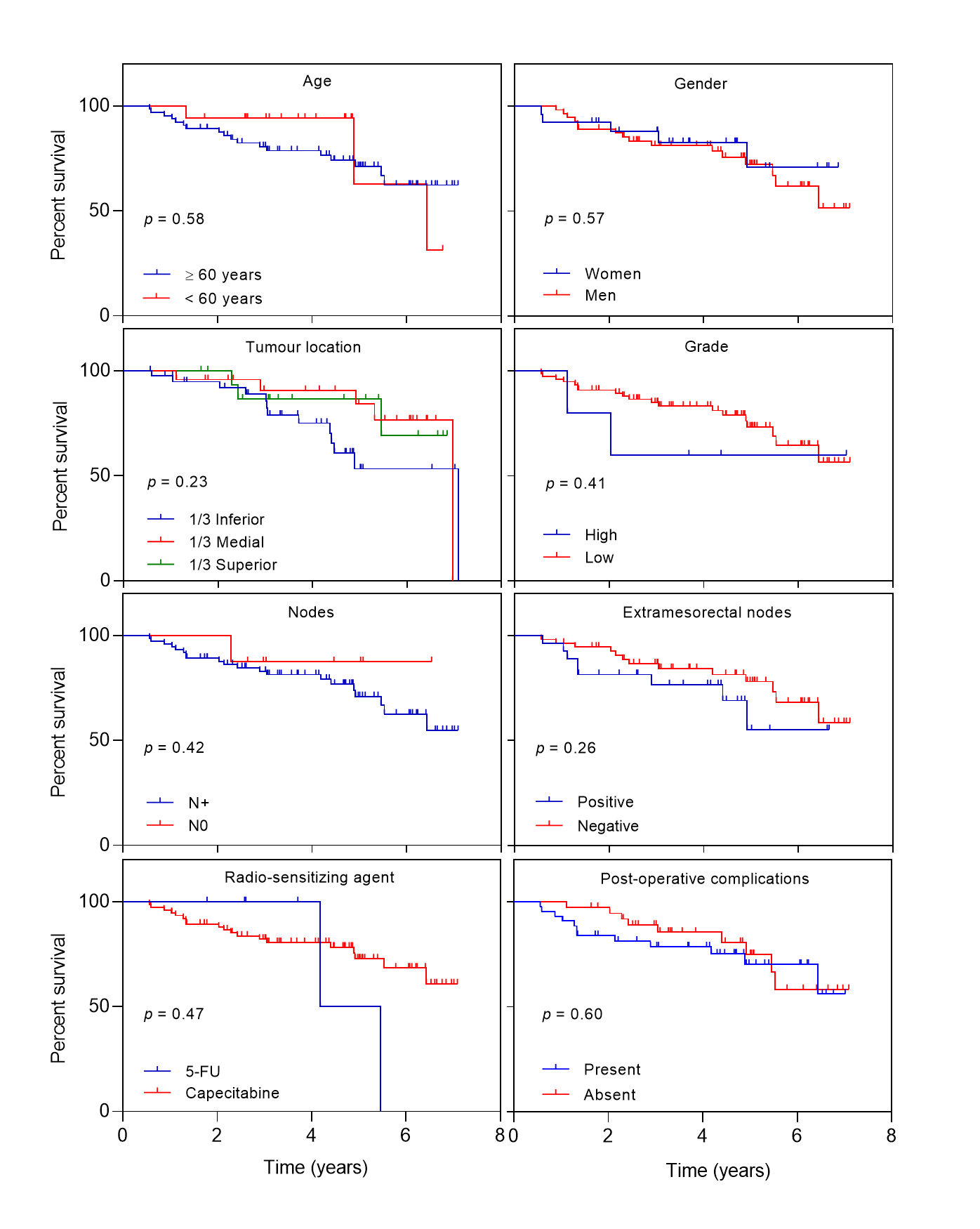


**Supplementary Figure S2.** Overall survival according to clinical and oncological parameters. Kaplan-Meier curves estimating overall survival according to age, gender, tumour location, grade, nodes, extramesorectal nodes, type of radio-sensitizing agent and post-operative morbidity.

**Table**

**Supplementary Table S1.** Preoperative CRT miR-21 predictive value.

|  | Incomplete response | | Complete response | | Total | |
| --- | --- | --- | --- | --- | --- | --- |
| miR-21 low | | 10 | | 24 | | 34 |
| miR-21 high | | 5 | | 43 | | 48 |
| Total | | 15 | | 67 | | 82 |
| PPV (%) 89.5 | |  | | Sensitivity (%) 64 | | |
| NPV (%) 29.4 | |  | | Specificity (%) 66 | | |

Cut-off derived by ROC curve.

miR-21 high: > 1.18; miR-21 low: < 1.18.

PPV: positive predictive value; NPV: negative predictive value.
